# Abnormal Cell Responses and Role of TNF-**α** in Impaired Diabetic Wound Healing

**DOI:** 10.1155/2013/754802

**Published:** 2013-01-20

**Authors:** Fanxing Xu, Chenying Zhang, Dana T. Graves

**Affiliations:** ^1^School of Life Science and Biotechnology, Dalian University of Technology, Dalian 116024, China; ^2^Department of Periodontics, School of Dental Medicine, University of Pennsylvania, Philadelphia, PA 19104, USA; ^3^Department of Preventive Dentistry, School and Hospital of Stomatology, Peking University, Beijing 100081, China

## Abstract

Impaired diabetic wound healing constitutes a major health problem. The impaired healing is caused by complex factors such as abnormal keratinocyte and fibroblast migration, proliferation, differentiation, and apoptosis, abnormal macrophage polarization, impaired recruitment of mesenchymal stem cells (MSCs) and endothelial progenitor cells (EPCs), and decreased vascularization. Diabetes-enhanced and prolonged expression of TNF-**α** also contributes to impaired healing. In this paper, we discuss the abnormal cell responses in diabetic wound healing and the contribution of TNF-**α**.

## 1. Introduction

Diabetes mellitus is one of the most prevalent and costly chronic diseases in the United States [1]. Impaired wound healing and diabetic foot ulcers constitute a major health problem in patients with diabetes. Diabetic foot ulceration is estimated to occur in 15% of diabetic patients, often requires prolonged hospitalization for its management, and is a major cause of disease-associated amputations in the western world [2].

**Figure 1:**
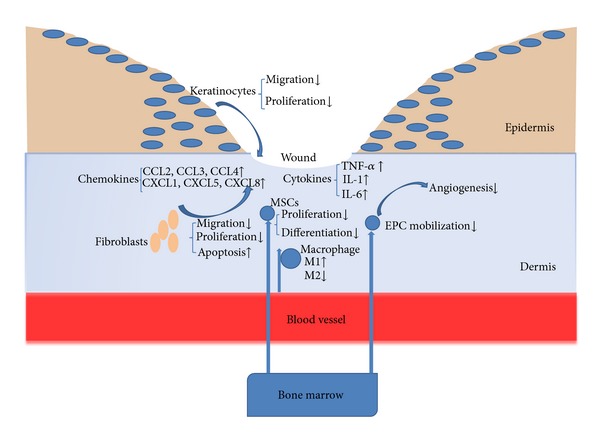
Mechanisms of impaired diabetic wound healing. The normal wound-healing process is initiated by the integration of multiple intercellular signals (cytokines and chemokines) released by keratinocytes, fibroblasts, endothelial cells, macrophages, platelets, etc. In diabetes, inflammatory cytokines and chemokines are elevated, such as TNF-*α*, IL-1, IL-6, CCL2, CCL3, CCL4, CXCL1, CXCL5, and CXCL8. Cellular processes affected by diabetes include abnormal keratinocyte and fibroblast migration, proliferation, and enhanced apoptosis; abnormal macrophage polarization (increased proinflammatory M1 macrophages and decreased anti-inflammatory M2 macrophages); impaired recruitment of mesenchymal stem cells (MSCs) and endothelial progenitor cells (EPCs), and decreased vascularization.

 Wound healing is a complex process involving a number of interdependent and overlapping stages including hemostasis, inflammation, proliferation, vascularization, and production of matrix and remodeling [3]. Many types of cells are involved in each phase of wound healing including immune cells, endothelial cells, keratinocytes, and fibroblasts which undergo marked changes in gene expression and phenotype [4, 5]. The delayed wound healing in diabetes is caused by complex factors such as diminished keratinocyte and fibroblast migration, proliferation, differentiation, apoptosis, and vascularization. Several of these cellular deficits have been linked to greater inflammation and proinflammatory cytokine production [6] (Figure 1).

Diabetic foot ulcers result from the simultaneous action of multiple contributing causes. A critical triad of neuropathy, minor foot trauma, and foot deformity is responsible for over 50% of diabetic foot ulcers [7]. Inflammation, immunodeficiency, peripheral neuropathy and ischemia from peripheral vascular disease, and subsequent infection are underlying factors that contribute to unhealed chronic wounds in diabetic foot ulcers [8].

One aspect of diabetic healing that has recently received considerable attention is the enhanced and prolonged expression of TNF-*α*, a potent proinflammatory cytokine [9].This review focuses on factors that are affected by diabetes-enhanced inflammation, particularly elevated or prolonged expression of TNF-*α*.

## 2. Cells Affected by Diabetes in Wound Healing

The inflammatory stage of wound repair occurs shortly after tissue damage. After acute injury, platelets and neutrophils are released passively from disrupted blood vessels. The formation of a fibrin clot provides a temporary scaffold for infiltration of inflammatory cells. A large number of growth factors are important in stimulating and coordinating cellular events that occur during normal wound healing [10]. Among them, cytokines and chemokines are especially noted because of their roles in promoting inflammation, angiogenesis, leukocyte recruitment, recruitment of stem cells, and epithelialization. Proinflammatory cytokines that are elevated shortly after wounding both in human wounds, and animal wound models include IL-1*α*, IL-1*β*, IL-6, IL-12, and TNF-*α* [11, 12]. Some proinflammatory cytokines and chemokines are essential for normal skin wound-healing process. Delayed wound healing is observed in IL-6-deficient mice [13]. It has been shown that deletion of IL-1 receptor signaling impairs oral wound healing due to its importance in upregulating an antibacterial defense but has relatively little impact on dermal healing [14]. The lack of ICAM-1 in mice results in prolonged wound healing because of the decreased recruitment of macrophages and other leukocytes [15, 16].

The CXC chemokine family of chemotactic cytokines CXCL1, CXCL5, and CXCL8 is expressed in keratinocytes and upregulated in wounding by stimulation of proinflammatory cytokines such as IL-1 and TNF-*α*, bacterial products, and hypoxia [17]. The induced expression of chemokines stimulates recruitment of leukocytes and monocytes, neutrophils, and macrophages to the wound site to remove foreign material, bacteria, dead cells, and damaged matrix [3]. Chemokine CX3CL1 and its receptor CX3CR1 were both highly induced at wound sites mediating recruitment of bone marrow-derived monocytes/macrophages in a mouse model of excisional skin wound healing [18]. CXCR3 chemokine receptor and its ligands CXCL11, CXCL10, and CXCL4 are also crucial for dermal maturation. Disruption of CXCR3 signaling in mice results in delayed reepithelialization [19]. Chemokines also induce recruitment of stem cells to sites of injury and include epithelial stem cells from hair follicles or sweat glands, endothelial progenitor cells, and mesenchymal stem cells [20–22]. 

Impaired wound healing in diabetic patients is accompanied by decreased early inflammatory cell infiltration but increased numbers of neutrophils and macrophages in late stages. These changes in inflammatory cell recruitment occur in conjunction with alterations in chemokine and growth factor expression [23]. An increase in inflammatory cytokines is observed in wounds of type-1 diabetic patients including CD40, IL-1*α*, IL-2, IL-4, IL-5, granulocyte-macrophage colony-stimulating factor (GM-CSF), CCL3, and CCL4 [24]. In diabetic models, increased levels of the proinflammatory cytokines such as TNF-*α* and IL-6 and decreased levels of anti-inflammatory IL-10 are observed in diabetic wound tissue compared to nondiabetic healing wound [25, 26]. This leads to sustained expression of chemokines CXCL2 and CCL2 that cause prolonged infiltration of leukocytes during impaired healing in diabetic mice [27].

### 2.1. Macrophages

Wound-site macrophages represent a key player that drives wound inflammation. Macrophages are important in clearance of dead cells and debris within the wound. Depletion of macrophages during the inflammatory phase results in significant delay of wound repair in a mouse model [28]. Diabetes is known to compromise macrophage function including phagocytosis activity [29]. Macrophages isolated from wounds of diabetic mice and diabetic patients showed significant impairment in efferocytosis, leading to a higher burden of apoptotic cells in wound tissue as well as increased proinflammatory cytokine expression [25]. 

High glucose levels stimulate macrophages to enhance the production of proinflammatory cytokines such as IL-1*β*, IL-6, IL-12, IL-18, TNF-*α*, and IFN-*γ* both *in vivo* and *in vitro* [12]. Macrophages may polarize along two lines that have functional differences, proinflammatory macrophages (M1), and anti-inflammatory macrophages (M2), which can be further subdivided in M2a (after exposure to IL-4 or IL-13), M2b (immune complexes in combination with IL-1*β* or LPS), and M2c (IL-10, TGF-*β* or glucocorticoids) [30]. M1 macrophages are polarized by the stimulation of IFN-*γ*, GM-CSF and in the presence of bacterial products such as LPS [30]. M1 macrophages have a proinflammatory phenotype exhibiting increased phagocytic activity and secretion of proinflammatory cytokines that aid in the removal of pathogens and damaged tissues [31, 32]. M2 macrophages have a polar opposite phenotype exhibiting high levels of anti-inflammatory cytokines and fibrogenic and angiogenic factors that serve to resolve inflammation and promote wound healing [30, 32]. Recently, an additional M2 subtype (M2d) which involves “switching” from an inflammatory M1 into an angiogenic M2 phenotype was discovered [33]. M2d macrophages express high levels of IL-10 and VEGF and low levels of TNF-*α* and IL-12 [33]. Macrophage polarization may play an important role in the pathogenesis of obesity-induced insulin resistance and type 2 diabetes mellitus [34]. Macrophages isolated from diabetic mice exhibit greater infiltration by inflammatory M1 macrophages and may contribute to impaired diabetic wound healing [35]. 

Wound macrophages in the early stage of repair are more M1-like when the generation of inflammatory signals is important while M2 macrophages predominate in later stages of repair in response to the need for new tissue formation [36]. In the normal wounds, the M1 macrophage phase is relatively short and the phase with M2 macrophages is longer [37]. M2 macrophages are a prominent source of TGF-*β*, which promotes many aspects of wound repair including chemotaxis, wound contraction, angiogenesis, reepithelialization, and connective tissue regeneration [5]. Diabetes may prolong the phase of M1 macrophage polarization. In addition infection in chronic wounds leads to prolonged M1 macrophage activation, which in turn can delay healing [38, 39]. 

### 2.2. Mesenchymal Stem Cells

Adult mesenchymal stem cells (MSCs) have the capacity for self-renewal and differentiating into a variety of mesenchymal cell lineages such as fibroblasts, osteoblasts, adipocytes, and chondrocytes. Increasing evidence shows that MSCs participate in the regeneration of skin in cutaneous wounds [40]. Hypoxia-inducible factor-1*α* (HIF-1*α*) and chemokines such as CCL2 facilitate MSC mobilization into the peripheral blood and to sites of wound healing [41, 42]. In addition to forming fibroblasts and myofibroblasts, MSCs also enhance wound healing through the secretion of mediators such as VEGF-*α*, IGF-1, EGF, keratinocyte growth factor, angiopoietin-1, stromal-derived factor-1, CCL3, CCL4, and erythropoietin [43, 44]. MSCs also play an important role in immunomodulation and are anti-inflammatory. MSCs inhibit the proliferation and activation of effector T cells, natural killer (NK) cells, dendritic cells (DCs), and macrophages by promoting the formation of anti-inflammatory regulatory T cells [45]. Thus there are multiple mechanisms through which MSCs can promote wound healing. 

Diabetes has detrimental effects on MSCs. Bone marrow-derived MSCs from diabetic rats have reduced proliferation and reduced myogenic differentiation [46]. The application of autologous MSCs improves healing of chronic diabetic foot ulcers [47]. Local application of MSCs to the wound sites improves wound healing in normal and diabetic mice, with increased reepithelialization, cellularity, and angiogenesis [43]. MSCs enhance diabetic wound healing by reducing inflammation, upregulating the expression of growth factors, and promoting the proliferation of fibroblasts and basal keratinocytes in diabetic rats [48].

### 2.3. Keratinocytes

Wound healing requires the transition of basal and suprabasal keratinocytes from a sedentary phenotype to a migratory and hyperproliferative phenotype. The reepithelialization process involves local keratinocytes at the wound edges and epithelial stem cells from hair follicles or sweat glands [49, 50]. Keratinocytes are a major source of growth factors such as TGF-*β*, VEGF, EGF, KGF, and TGF-*α* that stimulate fibrogenesis and angiogenesis in adjacent tissue [4, 51, 52]. Although there is no direct evidence that the proliferative activity of keratinocytes is affected in diabetes, migration is impaired [53, 54]. Keratinocytes at the chronic ulcer edge from diabetic patients have a reduced expression of migration markers [53, 55]. *In vitro* keratinocytes have reduced migration and proliferation capacities in high-glucose conditions [56].

### 2.4. Fibroblasts and Myofibroblasts

Fibroblasts are the primary source of extracellular matrix proteins such as collagen and fibronectin [57]. In diabetic oral and dermal wounds fibroblastshavedecreasedmigration, proliferation, and increased apoptosis [58–60]. The proliferation and migration of diabetic rat fibroblasts are suppressed when the cells are cultured in high-glucose containing media [60, 61]. Myofibroblasts are specialized fibroblasts that contribute to wound healing by producing extracellular matrix and by generating a contractile force to bring the edges of a wound together. The transition from fibroblasts to myofibroblasts is influenced by mechanical stress, endothelin-1, TGF-*β*, and cellular fibronectin (ED-A splice variant) [62, 63]. During acute wound healing in nondiabetic mice, mRNA levels for both TGF-*β* RI and TGF-*β* RII in wound tissue are elevated [64]. TGF-*β* receptor elevation is reduced in chronic diabetic ulcers [65]. Diabetics have reduced levels of TGF-*β* and reduced formation of myofibroblasts which may contribute to impaired wound contraction [66]. 

### 2.5. Endothelial Cells

Angiogenesis is a complex cascade of cellular, humoral, and molecular events, which initiates at the binding of growth factors to their receptors on the endothelial cells of existing vessels, such as VEGF. The stimulated endothelial cells proliferate and migrate into the wounded tissue to form small tubular canals which then mature [3]. Impaired angiogenesis is considered a major contributing factor to nonhealing wounds. Wound-induced hypoxia stimulates vascular regeneration by activating hypoxia-inducible transcription factors (HIF-1*α*), which increase the production of angiogenic growth factors such as VEGF and expression of the chemokine receptor CXCR4 [67]. The number and function of endothelial progenitors are reduced in diabetes mellitus [68, 69]. The importance of angiogenesis in contributing to impaired diabetic healing is demonstrated by improvement when diabetic wounds are treated with endothelial progenitors or VEGF. Injection of CD 34+ endothelial cell progenitors to the wounds of diabetic mice accelerates vascularization and healing of diabetic mouse skin wounds [70]. Topical application of VEGF also improves diabetic wound healing by locally upregulating growth factors PDGF and FGF-2 and promoting angiogenesis [71]. 

### 2.6. MMPs/TIMPs Imbalance in Diabetes

The balance between matrix metalloproteinases (MMPs) and tissue inhibitors of metalloproteinases (TIMPs) is crucial for normal wound healing processes. A low MMP/TIMP ratio is a good predictor of successful wound-healing in diabetic foot ulcers [72]. Diabetes creates an unfavorable ratio. It increases the activity and expression of MMP-9, MMP-2, and MMP-8 while reducing TIMP-2 [73, 74]. The abnormally elevated level of MMPs may impair cell migration and result in sustained inflammation with net increased tissue destruction. In the chronic diabetic foot lesions, local administration of protease inhibitors reduces the ratio of MMP/TIMP and improves wound healing [68]. 

## 3. Role of TNF-*α* in Diabetic Wounds

In normal wound healing the highest levels of TNF-*α* are seen from 12 to 24 h after wounding [75]. After the completion of the proliferative phase of wound healing, TNF-*α* returns to basal levels. During the early phase of wound repair, it is predominantly expressed in polymorphonuclear leukocytes, and later by macrophages. It is also expressed in the hyperproliferative epithelium at the wound edge. TNF-*α* contributes to the stimulation of fibroblasts and keratinocytes the expression of growth factors and upregulation of antimicrobial defenses [76]. TNF-*α* levels are elevated in diabetes in part through increased oxidative stress that promotes inflammation [77]. Other factors may contribute to this elevation including the downregulation of CD33 that inhibits cytokine production [78]. TNF-*α* is found threefold higher in diabetic mouse wounds than wounds in normal mice [59] and threefold higher found in wound fluid from nonhealing venous leg ulcers than in healing ulcers [79]. Chronic gastric ulcers are also associated with increased TNF-*α* [80].

### 3.1. Cellular Events Affected by TNF-*α*

**Figure 2:**
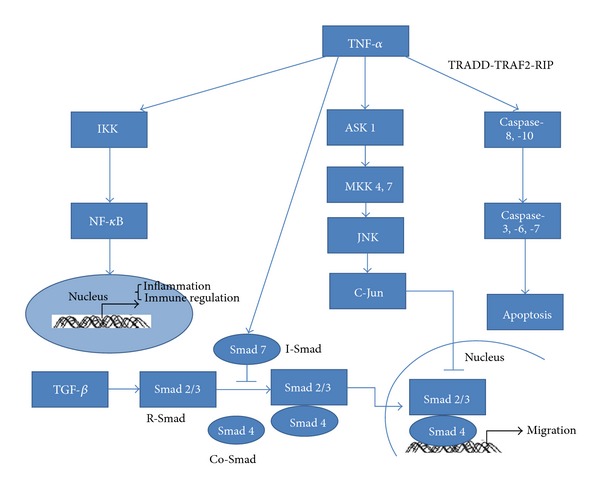
Model of TNF-*α* on regulation of inflammation, immue response, migration, and differentiation. TNF-*α* induces cell apoptosis via caspase pathway, and it negatively regulates cell migration by increasing the level of Smad 7 and inhibiting the activation of the Smad 2/3. TNF-*α* also induces NF-*κ*B activation to enhance inflammatory responses.

In diabetic wound healing impaired fibroblast proliferation has been linked to increased levels of TNF-*α* [81]. Inhibiting TNF *in vivo* significantly increases the number of proliferating fibroblasts but it has a little effect on fibroblast proliferation in normoglycemic mice [59]. Apoptosis of fibroblasts in diabetic mice is significantly higher than in normoglycemic counterparts [59, 82], and apoptosis is high in skin biopsies from diabetic foot ulcers [83, 84]. TNF stimulates apoptosis of fibroblasts, keratinocytes, and endothelial cells *in vitro* [85, 86]. A cause-and-effect relationship has been established between the treatment of TNF blocker and reduced apoptosis which was elevated in diabetic healing [59]. Diabetes also impairs the migration of fibroblasts and keratinocytes [55, 87]. High levels of TNF-*α* inhibit cell migration [88]. This may occur by increasing the level of Smad 7 [89] and inhibiting the activation of the Smad 2/3 [90] (Figure 2).

The neutralization of TNF in the diabetic wounds improves wound angiogenesis and closure. Blocking TNF reduces the overproduction of small noncoding RNAs such as miR-200b in the diabetic wounds, which improves the expression of globin transcription factor-binding protein 2 (GATA2) and vascular endothelial growth factor receptor 2 (VEGFR2), both of which promote angiogenesis [91].

The ability of cells at the wound site to respond to insulin is reduced in diabetic wounds. Insulin insensitivity occurs when the response to insulin is reduced. Long-term treatment of cells with TNF-*α* contributes to reduced insulin sensitivity [92]. Insulin receptor expression in proliferating keratinocytes at the wound margins and in granulation tissue is reduced in diabetic mice but enhanced with anti-TNF-*α* antibody treatment [93]. The effect of neutralization of TNF-*α* on insulin sensitivity may be involved in inhibiting the effects of TNF-*α* on the downregulation of GLUT4 genes that are required for normal insulin action, the downregulation of PPAR*γ* which is an important insulin-sensitizing nuclear receptor, and the upregulation of Ser phosphorylation of IRS-1 that results in a net decrease in insulin receptor-mediated signaling [94]. Thus, an important component of impaired diabetic wound healing may be due to the reduced sensitivity of cells that participate in the wound healing process to insulin stimulation, which is mediated in part by high levels of TNF.

### 3.2. Effect of TNF-Induced FOXO1 on Diabetic Wound Healing

Some of the negative effects of diabetes-enhanced TNF on wound healing may be due to the impact of the FOXO1 transcription factor [77, 95]. FOXO1 activity is increased in a number of different diabetic conditions and may be detrimental because it induces cell cycle arrest and apoptosis and increases the production of proinflammatory cytokines [77]. TNF-*α*-induced apoptosis of endothelial cells and pericytes is FOXO1 dependent *in vivo* and *in vitro* [96, 97]. *In vivo*, FOXO1 DNA activity is increased twofold in diabetic wounds, and the increase is driven by diabetes-enhanced TNF levels [59]. FOXO1 activity is also increased *in vivo *in fracture healing and linked to greater inflammation [98, 99]. In normal wound healing FOXO1 may play a positive role in endothelial migration and tube formation [95].

### 3.3. Advanced Glycation Endproducts

AGEs are proteins or lipids that become glycated after exposure to sugars. Enhanced formation and accumulation of advanced glycation end-products (AGEs) and receptors for AGEs have been reported to occur in diabetes mellitus [100–102]. The activation of one of the AGE receptors, (receptor for AGEs), RAGE causes the upregulation of the transcription factor nuclear factor-kappa B (NF-kappa B) and its target genes such as intercellular adhesion molecule-1 (ICAM-1), VEGF, IL-1*α*, IL-6, and TNF-*α*. Mice fed with high levels of AGE display impaired wound closure [103]. Blockade of RAGE restores effective wound healing in diabetic mice by accelerating reepithelialization and angiogenesis, limiting inflammatory cell infiltration, and reducing the expression of TNF-*α*, IL-6, MMP-2, -3, and -9 [100]. AGEs cause the production of reactive oxygen species at least in part, through the activation of NADPH oxidase [77, 104]. In mononuclear phagocytes, AGEs increases the generation of cytokines such as TNF-*α*, IL-1, and IL-6 and enhanced the production of O^2−^ [101, 105, 106].

## 4. Conclusion 

The impaired diabetic wound healing and diabetic ulcer impair the quality of life of millions of people and burden the healthcare systems globally. The etiological factors involve a high level of TNF-*α*, which inhibits angiogenesis and cell proliferation and migration in diabetic wounds and increases apoptosis levels. TNF inhibition attenuates the impact of diabetes-enhanced TNF-*α*, which offers potentially new therapeutic avenue for treatment of abnormally diabetic wounds healing.
